# Functional Outcomes after Surgical Decompression for Tarsal Tunnel Syndrome

**DOI:** 10.1055/s-0046-1819577

**Published:** 2026-04-17

**Authors:** Marco Antonio Casares Tamayo, Jonathan Antonio Casares Castellanos, Francisco Endara Urresta, Carlos Peñaherrera Carrillo, Alejandro Barros Castro

**Affiliations:** 1 Department of Orthopedics and Traumatology, Hospital Metropolitano, Quito, Ecuador; 2 Pontificia Universidad Católica del Ecuador, Quito, Ecuador; 3 Clínica Arthros, Quito, Ecuador; 4 Instituto Nacional de Rehabilitación Luis Guillermo Ibarra Ibarra (LGII), Universidad Autónoma de México, Mexico City, Mexico; 5 Department of Orthopedics and Traumatology, Universidad Internacional del Ecuador, Quito, Ecuador

**Keywords:** decompression, electromyography, tarsal tunnel syndrome, ultrasonography, decompressão, eletromiografia, síndrome do túnel do tarso, ultrassonografia

## Abstract

**Objectives:**

To evaluate the functional outcomes of surgical decompression for tarsal tunnel syndrome (TTS) and to explore the clinical relevance of diagnostic tests such as electromyography (EMG), magnetic resonance imaging (MRI), and ultrasound.

**Methods:**

We performed a retrospective single-center study of 15 patients with clinically diagnosed TTS who underwent open decompression (2015–2022). All had failed conservative management and completed ≥ 12 months of follow-up. Preoperative evaluation combined clinical exams with at least one confirmatory test (EMG, MRI, or ultrasound). Outcomes included the American Orthopaedic Foot and Ankle Society's (AOFAS) ankle–hindfoot score (primary), Visual Analogue Scale (VAS) pain, minimal clinically important difference (MCID), and complications. The analysis used paired *t* -tests, Cohen's d, and analysis of covariance (ANCOVA), adjusting for baseline AOFAS, age, symptom duration, and EMG results.

**Results:**

The mean age was 50.4 ± 15.6 years; and 53.3% of the patients were female. Symptom duration averaged 14.7 ± 7.2 months. The EMG scans were positive in 66.7%, MRI in 60.0%, and ultrasound in 53.3%. The AOFAS score improved from 36.6 ± 7.1 to 78.1 ± 19.9 at 12 months ( *p* < 0.001; d = 2.26), and VAS decreased from 7.0 ± 0.6 to 3.4 ± 1.3 ( *p* < 0.001; d = −3.72). The MCID was achieved in 80% for AOFAS and 100% for VAS. Two minor complications (13.3%) occurred, without need for reoperations. The ANCOVA suggested trends for baseline severity and chronicity without significance.

**Conclusion:**

Surgical decompression is a safe and effective treatment for TTS, providing significant pain relief and functional improvement with low complication rates. Early intervention shows a trend toward better outcomes, warranting further prospective studies.

## Resumo

**Objetivos:**

Avaliar os desfechos funcionais da descompressão cirúrgica para tratamento da síndrome do túnel do tarso (STT) e explorar a relevância clínica de exames diagnósticos como eletromiografia (EMG), ressonância magnética (RM) e ultrassonografia (US).

**Métodos:**

Realizamos um estudo retrospectivo unicêntrico com 15 pacientes com diagnóstico clínico de STT submetidos à descompressão cirúrgica aberta (2015–2022). O tratamento conservador falhou em todos, sendo acompanhados por pelo menos 12 meses. A avaliação pré-operatória combinou o exame clínico com pelo menos um exame confirmatório (EMG, RM ou US). Os desfechos incluíram o escore (primário) da American Orthopaedic Foot and Ankle Society (AOFAS) para tornozelo e retropé, dor avaliada pela Escala Visual Analógica (EVA), diferença mínima clinicamente importante (DMCI) e complicações. A análise utilizou testes t pareados, d de Cohen e análise de covariância (ANCOVA), com ajuste conforme o escore AOFAS basal, idade, duração dos sintomas e resultados da EMG.

**Resultados:**

A idade média foi de 50,4 ± 15,6 anos; e 53,3% dos pacientes eram do sexo feminino. A duração média dos sintomas foi de 14,7 ± 7,2 meses. A EMG foi positiva em 66,7% dos casos, a RM em 60,0%, e a US em 53,3%. O escore AOFAS melhorou de 36,6 ± 7,1 para 78,1 ± 19,9 em 12 meses ( *p* < 0,001; d = 2,26) e a EVA diminuiu de 7,0 ± 0,6 para 3,4 ± 1,3 ( *p* < 0,001; d = −3,72). A DMCI foi alcançada em 80% dos casos para o escore AOFAS e em 100% para EVA. Ocorreram duas complicações menores (13,3%); não houve necessidade de nova intervenção cirúrgica. A ANCOVA sugeriu tendências de gravidade basal e cronicidade, mas sem significância estatística.

**Conclusão:**

A descompressão cirúrgica é um tratamento seguro e eficaz para a STT, proporcionando alívio significativo da dor e melhora funcional com baixas taxas de complicações. A intervenção precoce tende a gerar desfechos melhores, o que justifica a realização de mais estudos prospectivos.

## Introduction

Tarsal tunnel syndrome (TTS) is an entrapment neuropathy of the posterior tibial nerve or its branches beneath the flexor retinaculum at the medial aspect of the ankle. Its etiology is multifactorial, encompassing extrinsic causes such as trauma, hindfoot deformity, or varicosities, as well as intrinsic factors including synovial cysts, ganglia, muscular hypertrophy, or fibrotic changes. Clinically, the typical presentation is burning pain in the hindfoot and plantar aspect of the foot, paresthesias, and, in more advanced cases, intrinsic muscle weakness or atrophy. The functional impact of TTS is considerable, as it limits prolonged ambulation, induces intolerance to standing, and reduces health-related quality of life, affecting both younger active individuals and older adults. 1 2 3

Diagnosis of TTS remains challenging. Clinical examination may reveal a positive Tinel's sign over the flexor retinaculum or reproduce symptoms with provocative maneuvers; however, the sensitivity and specificity of these techniques are inconsistent. Ancillary tests are frequently employed to objectify the presence of nerve compression, though their diagnostic performance varies considerably.

Electromyography (EMG) is the most widely used tool to document neurophysiological compromise, yet reported sensitivity ranges from 45 to 80%, with false negatives being particularly common in the early stages of disease. Magnetic resonance imaging (MRI) can identify secondary causes such as space-occupying lesions or anatomical variants, but it does not always capture dynamic compression. Musculoskeletal ultrasound has been increasingly preferred as it provides real-time visualization of the tibial nerve and adjacent structures, and allows for dynamic assessment; nevertheless, its diagnostic accuracy is operator-dependent. This heterogeneity across diagnostic modalities contributes to uncertainty and often delays the indication for surgery. 4 5 6

Conservative treatment strategies—including orthoses, corticosteroid injections, and physiotherapy—may provide temporary symptomatic relief. However, in patients with persistent symptoms and functional limitation, surgical decompression of the tarsal tunnel remains the treatment of choice. Published case series have reported clinical success rates ranging between 70 and 90%, with significant improvement in both pain and function at mid-term follow-up. Nonetheless, heterogeneity in diagnostic criteria, surgical technique, and outcome measures hampers comparability across studies, and little is known about the patient- and disease-related factors that modulate surgical outcomes. 7

Against this background, the present study aims to systematically evaluate the functional outcomes of surgical decompression for TTS in a consecutive cohort of patients, using the American Orthopaedic Foot and Ankle Society (AOFAS) score as the primary endpoint. Secondary outcomes include pain reduction measured by the Visual Analogue Scale (VAS), attainment of minimal clinically important difference (MCID), and complication rates. Additionally, we sought to explore the relative diagnostic yield of EMG, MRI, and ultrasound, to provide practical evidence regarding their utility in clinical decision-making.

As such, the study had two main objectives: to evaluate functional outcomes (AOFAS, VAS, MCID, complications) after surgical decompression for TTS; and to explore the clinical relevance of ancillary diagnostic tests (EMG, MRI, ultrasound) in supporting diagnosis and guiding surgical indication.

## Methods

This investigation was designed as a single-center retrospective cohort study performed at a tertiary referral institution with a dedicated foot and ankle unit. The study protocol was conducted in accordance with institutional ethical standards, adhering to the principles of the Declaration of Helsinki.

The study population consisted of 15 consecutive patients diagnosed with TTS who underwent surgical decompression between January 2015 and December 2022. Inclusion criteria were adults older than 18 years with a clinical diagnosis of TTS characterized by medial ankle or plantar foot pain, paresthesias, or neurological deficit consistent with tibial nerve entrapment; and who had failed conservative management for at least 3 months. The conservative treatment included changes in activity, rest, and use of nonsteroidal anti-inflammatory drugs, in association with daily physical therapy emphasizing stretching and nerve-gliding exercises, which can improve flexibility and minimize entrapment.

Patients were required to complete baseline and follow-up clinical data, including standardized outcome scores. Exclusion criteria comprised previous hindfoot surgery, systemic neuropathies (i.e. diabetic polyneuropathy), inflammatory arthropathies, or incomplete clinical records.

All patients underwent a uniform preoperative evaluation that combined detailed clinical examination with at least one confirmatory ancillary test. Clinical assessment included inspection, palpation along the flexor retinaculum, and provocative maneuvers, such as Tinel's sign or dorsiflexion-eversion testing. Furthermore, EMG scan was performed in all the patients to document neurophysiological impairment of the tibial nerve. EMG results were positive in ten patients. Subsequently, a musculoskeletal ultrasound was performed in the ten patients with ambiguous symptoms to dynamically assess the tibial nerve and surrounding structures—such as plantar pain, paresthesia, burning in the heel or sole, or mild/inconsistent neurological signs—or in those with suspected space-occupying lesions or prior trauma potentially causing scarring; the scan showed abnormalities in eight patients. Finally, an MRI was performed in these eight patients to confirm space-occupying lesions previously identified on ultrasound and one patient with prior trauma, revealing the suspected pathologies. The combination of these modalities was employed to reduce diagnostic uncertainty and to guide surgical indication.

Surgical intervention consisted of an open decompression of the tarsal tunnel performed under regional or general anesthesia by the same surgical team with a medial approach. Intraoperative findings included perineural edema, synovitis, and flexor retinaculum thickening (incised longitudinally, with careful release of the posterior tibial nerve and its branches) in 15 patients (100%). Additionally, 8 patients (53.3%) had space-occupying lesions (synovial cysts), which were resected; and 2 patients (13.3%) showed perineural fibrosis and scarring, which were removed. Hemostasis was meticulous, and the wound was closed in layers without tension. No adjunctive procedures, such as neurolysis beyond the retinacular level or tendon transfer, were performed in this series.

Postoperatively, all patients followed a standardized rehabilitation protocol. The ankle was immobilized for 2 weeks in a neutral position, after which weight-bearing was gradually resumed as tolerated. Physiotherapy focused on neural gliding exercises, control of perineural edema, and progressive strengthening of intrinsic and extrinsic musculature. Patients were followed clinically at regular intervals up to 12 months after surgery.

The primary outcome of interest was the change in the AOFAS ankle–hindfoot score from baseline to 12 months. Secondary outcomes included pain intensity measured by the VAS, attainment of the MCID defined as an improvement of ≥ 10 points in the AOFAS ankle–hindfoot score and ≥ 2 points in the VAS for pain. These thresholds are consistent with previously published MCID values for foot and ankle surgery, where changes of 8 to 12 points in AOFAS and 1.5 to 2.0 points in VAS have been shown to represent perceptible and clinically meaningful improvements for patients with hindfoot or tarsal tunnel pathologies, 8 9 10 as well as the incidence of perioperative complications and the need for reoperation.

Statistical analysis was performed using standard methods for paired data. Continuous variables were summarized as mean and standard deviation (SD) or median and interquartile range (IQR), as appropriate. Categorical variables were expressed as absolute numbers and percentages. The paired *t* -test was selected to compare pre- and postoperative AOFAS and VAS scores because the same individuals were evaluated at two time points, and the test efficiently assesses mean changes in continuous outcomes.

Although the sample size was modest ( *n* = 15), the *t* -test is generally robust to mild departures from normality, particularly when the data are symmetrically distributed. Prior to analysis, the normality of the difference scores was assessed using the Shapiro–Wilk test and inspection of Q–Q plots; both confirmed approximate normal distribution, supporting the use of parametric testing. Nevertheless, a sensitivity analysis using the non-parametric Wilcoxon signed-rank test yielded comparable significance levels, confirming the robustness of the findings. Effect sizes were calculated using Cohen's d to quantify the magnitude of change.

The MCID attainment rates were calculated as proportions. To explore predictors of functional outcome, an analysis of covariance (ANCOVA) was conducted with postoperative AOFAS as the dependent variable and baseline AOFAS, patient age, symptom duration, and EMG positivity as covariates. A two-tailed *p* -value < 0.05 was considered statistically significant.

## Results

A total of 15 consecutive patients fulfilled the eligibility criteria and were available for analysis after completing follow-up of 12 months. These baseline characteristics are summarized in Table 1 .

**Table 1:** Baseline characteristics of the study cohort

Variable	Value (n = 15)
Age, years (mean ± SD)	50.4 ± 15.6
Female sex, n (%)	8 (53.3)
Laterality: Left / Right, n	7/8
Symptom duration, months (mean ± SD)	14.7 ± 7.2
Positive EMG, n (%)	10 (66.7)
Positive MRI, n (%)	9 (60.0)
Positive ultrasound, n (%)	8 (53.3)

**Abbreviations:** EMG, electromyography; MRI, magnetic resonance imaging; SD, standard deviation.

Postoperative evaluation revealed statistically and clinically significant improvements in functional and pain outcomes. The mean AOFAS ankle–hindfoot score increased from 36.6 ± 7.1 preoperatively to 78.1 ± 19.9 at the 12-month follow-up, corresponding to a mean gain of 41.5 ± 18.4 points. This difference was highly significant (t = 8.74, *p* < 0.001) and associated with a large effect size (Cohen's d = 2.26), supporting the robustness of the functional benefit observed. Pain intensity, as assessed by the VAS, decreased from 7.0 ± 0.6 at baseline to 3.4 ± 1.3 at the latest follow-up, reflecting a mean reduction of −3.6 ± 1.0 points (t = −14.43, *p* < 0.001; Cohen's d = −3.72). The proportion of patients achieving the MCID was 80.0% for AOFAS and 100.0% for VAS, indicating that improvements were not only statistically significant but also clinically meaningful for the majority of individuals. Detailed outcomes are provided in Table 2 .

**Table 2:** Functional outcomes before and after surgical decompression

Outcome	Preoperative (mean ± SD)	12 months postoperative (mean ± SD)	Mean difference (±SD)	*p* -value	MCID (%)
AOFAS score	36.6 ± 7.1	78.1 ± 19.9	41.5 ± 18.4	< 0.001	80.0
VAS pain	7.0 ± 0.6	3.4 ± 1.3	−3.6 ± 1.0	< 0.001	100.0

**Abbreviations:** AOFAS, American Orthopaedic Foot and Ankle Society; MCID, minimal clinically important difference; SD, standard deviation; VAS, visual analogue scale.

To investigate potential predictors of functional recovery, an ANCOVA model was applied using the postoperative AOFAS score as the dependent variable, adjusting for the baseline score, age, symptom duration, and EMG status. Although higher baseline scores and longer duration of symptoms showed trends toward influencing final functional results, none of the covariates reached statistical significance in this sample. This finding may reflect the modest cohort size and associated limitations in statistical power. The ANCOVA model results are presented in Table 3 .

**Table 3:** The ANCOVA model for predictors of postoperative AOFAS ( *n* = 15)

Covariate	β coefficient	*p* -value
AOFAS baseline	1.08	0.159
Age	0.33	0.465
Symptom duration (months)	−1.22	0.152
EMG positivity	−11.83	0.384

**Abbreviations:** AOFAS, American Orthopaedic Foot and Ankle Society; EMG, electromyography.

**Fig. 1:**
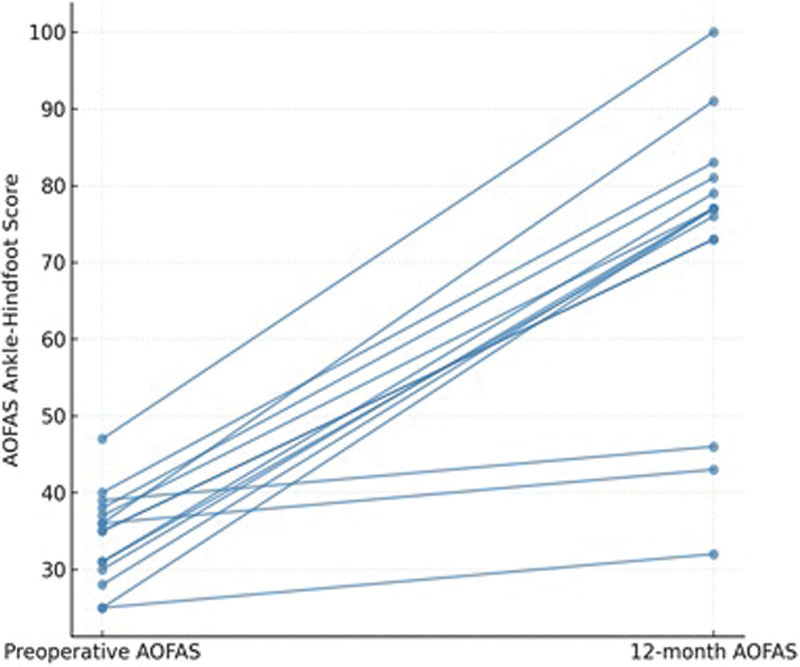
Distribution of individual American Orthopaedic Foot and Ankle Society (AOFAS) scores before and after surgical decompression, highlighting the consistent improvement observed across the cohort.

There were three postoperative complications documented (20.0%), including two cases of transient paresthesia and one superficial wound problem. They were all managed conservatively and resolved without sequelae. This is probably the reason why the postoperative AOFAS score is below 50. Importantly, no patient required reoperation during the follow-up period ( Fig. 1 ).

## Discussion

Our findings are consistent with prior series reported in the literature, in which success rates following surgical release range between 70 and 90%. These studies, conducted in heterogeneous populations with varying diagnostic protocols and surgical techniques, have generally demonstrated meaningful improvements in both pain and functional outcomes. The magnitude of benefit observed in the current cohort aligns with the upper range of previously published reports, further validating the effectiveness of decompression when appropriately indicated. The relatively low incidence of complications and absence of revision procedures in this series also compare favorably with existing evidence, which typically cites minor wound issues or transient neurological symptoms as the most common adverse events. 11 12 13

A distinctive feature of this study is the systematic integration of ancillary diagnostic modalities into the preoperative assessment and their subsequent analysis in relation to outcomes. The EMG, MRI, and ultrasound scans were variably positive across the cohort, reflecting the heterogeneity and imperfect sensitivity of each tool. The exploratory modeling using ANCOVA, while underpowered due to sample size, suggested that baseline AOFAS and symptom duration may influence postoperative recovery. This trend may be interpreted as a marker of more advanced or chronic neuropathic involvement, in which structural and physiological changes are less reversible even after adequate decompression. Such observations are clinically relevant, as they highlight the importance of not delaying surgical referral once conservative measures have failed and symptoms persist beyond a reasonable time frame. 14 15 16

The postoperative mean AOFAS score of 78.1 and VAS of 3.4 observed in our cohort indicate a substantial functional and symptomatic improvement, consistent with clinically meaningful recovery after decompression. These results fall within the range reported in previous series, where postoperative AOFAS scores typically range from 70 to 85 and VAS values between 2 and 4, being associated with good to excellent outcomes. For example, Lalevée et al. 12 reported a mean postoperative AOFAS of 80.6 following open tarsal tunnel release, while Rungprai et al. 4 observed similar gains regardless of preoperative electrodiagnostic status. Likewise, Iborra et al. 2 and Sun et al. 11 described postoperative pain scores around 3 on the VAS, correlating with high patient satisfaction and restoration of daily activities. These findings suggest that the functional recovery achieved in the present series meets established benchmarks of clinical success for tarsal tunnel decompression. Minor residual symptoms or incomplete improvement in some patients likely reflect chronicity of nerve compression or preexisting neuropathic changes, phenomena also reported in the literature. 8 9 10 16 17 18

From a clinical standpoint, these results emphasize two important implications. First, timely surgical intervention may maximize functional recovery by preventing irreversible neural damage associated with prolonged compression. Second, the judicious use of diagnostic tests should be tailored to the clinical context: EMG remains valuable in documenting neurophysiological compromise; MRI is particularly useful for excluding secondary causes, such as ganglia or space-occupying lesions; and ultrasound offers dynamic assessment that can complement static imaging. The combination of these modalities, interpreted in the context of a thorough clinical examination, allows for a more precise selection of surgical candidates. 17 18 19 20

This study has several limitations that must be acknowledged. The sample size was relatively small, limiting the statistical power to detect subtle predictors of outcome and precluding robust multivariate analysis. The retrospective design introduces the possibility of selection bias and relies on the completeness of medical records. Additionally, the absence of a control group receiving nonoperative treatment or sham surgery prevents direct comparison of surgical efficacy against conservative management. Follow-up was limited to a minimum of 12 months, and longer-term data are needed to determine the durability of clinical benefit. 21 22 23

## Conclusion

Surgical decompression is a safe and effective treatment for TTS refractory to conservative care, providing meaningful improvements in pain and function with few complications. Early intervention shows a trend toward better outcomes, but larger prospective studies are needed to confirm prognostic factors and clarify the diagnostic value of EMG, MRI, and ultrasound.
